# Identification of genes for complex disease using longitudinal phenotypes

**DOI:** 10.1186/1471-2156-4-S1-S58

**Published:** 2003-12-31

**Authors:** Nathan Pankratz, Nitai Mukhopadhyay, Shuguang Huang, Tatiana Foroud, Sandra Close Kirkwood

**Affiliations:** 1 Medical and Molecular Genetics, Indiana University School of Medicine, Indianapolis, Indiana, USA; 2 Eli Lilly and Company, Indianapolis, Indiana, USA

## Abstract

Using the simulated data set from Genetic Analysis Workshop 13, we explored the advantages of using longitudinal data in genetic analyses. The weighted average of the longitudinal data for each of seven quantitative phenotypes were computed and analyzed. Genome screen results were then compared for these longitudinal phenotypes and the results obtained using two cross-sectional designs: data collected near a single age (45 years) and data collected at a single time point. Significant linkage was obtained for nine regions (LOD scores ranging from 5.5 to 34.6) for six of the phenotypes. Using cross-sectional data, LOD scores were slightly lower for the same chromosomal regions, with two regions becoming nonsignificant and one additional region being identified. The magnitude of the LOD score was highly correlated with the heritability of each phenotype as well as the proportion of phenotypic variance due to that locus. There were no false-positive linkage results using the longitudinal data and three false-positive findings using the cross-sectional data. The three false positive results appear to be due to the kurtosis in the trait distribution, even after removing extreme outliers. Our analyses demonstrated that the use of simple longitudinal phenotypes was a powerful means to detect genes of major to moderate effect on trait variability. In only one instance was the power and heritability of the trait increased by using data from one examination. Power to detect linkage can be improved by identifying the most heritable phenotype, ensuring normality of the trait distribution and maximizing the information utilized through novel longitudinal designs for genetic analysis.

## Background

Studies designed to identify genes contributing to complex disease have been ongoing for many years, utilizing assorted study designs and analysis methods with varied success. The Genetic Analysis Workshop 13 (GAW13) simulated data set provides an opportunity to explore the advantages of longitudinal as compared with cross-sectional study designs. For each univariate phenotype, we compared linkage results generated from a weighted average of the longitudinal data with results from two different cross sectional designs: data gathered at a single examination and data obtained from one examination collected during the age range (mid-40s) when the phenotypes were most heritable.

## Methods

All analyses were performed using the simulated data set without knowledge of the underlying model, including number of genes or their location. Analyses were performed with complete genotypic and phenotypic data from Replicate 1. The true model was obtained only at GAW13, and was used in this manuscript to identify true and false positives.

### Phenotype development

The simulated data set included a number of cardiovascular phenotypes, blood pressure, lipid, and glucose measurements, which were collected longitudinally in the study subjects. Unfortunately, data were gathered at different intervals in the first and second cohort. In addition, the subjects in the two cohorts participated in the study at variable ages. To extract maximal informativeness for linkage studies, we developed phenotypes that were both heritable and capitalized on the longitudinal phenotypic information.

A univariate phenotype was calculated for each of the measures: body mass index (BMI), total cholesterol (CHOL), fasting glucose (GLUC), high-density lipoprotein (HDL), height (HEIGHT), systolic blood pressure (SBP), and triglycerides (TG). Longitudinal data was provided for these traits at different time points and with differing amounts of time between consecutive measurements. The area under the curve obtained by joining consecutive measurements over the time scale capitalized on the longitudinal nature of the data, yet accounted for the non-uniform amount of time between consecutive measurements [1]. This area, divided by the duration of observation, is simply a weighted average of the phenotype measurements with weights proportional to the time difference between the previous measurement and the current measurement. This derived univariate measure is referred to as AUC in this paper.

**Figure 1:**
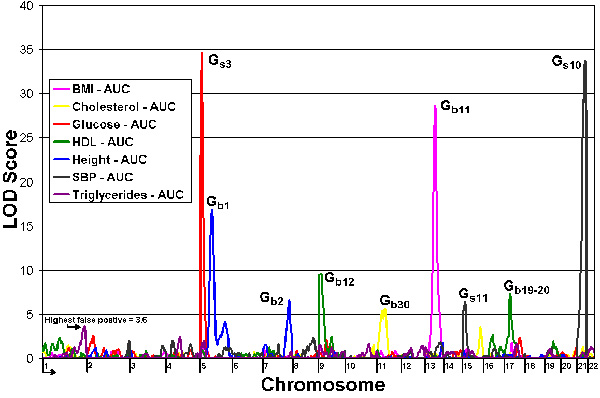
**Genome screen results for longitudinal data (AUC) **All 2860 individuals were used in all of the longitudinal analyses.

Data points were also selected to simulate a cross-sectional study design (single exam; SE). Exam 12 was selected for Cohort 1, since it was the only patient visit where all phenotypes were collected (except height, which was taken from exam 10). Exam 1 was selected for Cohort 2. Another cross-sectional design selected data points to simulate a study design that collected people in their mid-40s (AGE = 45). The patient visit closest to age 45, and within 5 years, was selected for each individual for each phenotype. The age of 45 was chosen because data were available for the largest number of individuals (2674; other ages ranges only had 763 to 2160 individuals) and the phenotypes tended to be highly heritable at that age (0.71–0.81 versus 0.56–0.85). For these cross-sectional study designs, the number of individuals with available data was smaller than that for the longitudinal design. So, to make a valid comparison between the two methods, an additional set of longitudinal phenotypes were calculated for each of the cross-sectional designs that utilized the same individuals. (These additional sets were only used as a comparison in Tables 2 and 3; all 2860 individuals were used in the analyses summarized in Table 1 and Figure 1.)

Since deviation from normality is known to increase the false-positive rate of certain genetic analyses, careful attention was paid to the trait distribution of each phenotype. GLUC and TG were particularly kurtotic, and so we systematically removed trait values from all analyses that were in excess of three standard deviations from the mean. Since these outliers might represent true extremes caused by genetic effects, we considered truncating the data instead of removing them. However, this would not address the kurtosis that is known to cause false-positive results using Sequential Oligogenic Linkage Analysis Routines (SOLAR) [2], and so we decided to err on the side of specificity.

### Genetic Analysis

Heritability estimates were calculated for each of the phenotypes using SOLAR. Multipoint linkage analysis was performed for all seven phenotypes (AUC of BMI, CHOL, GLUC, HDL, HEIGHT, SBP, and TG) for each of the three study designs (AUC, SE, AGE = 45) using the pedigree-based variance component method implemented in the program SOLAR with simultaneous correction for [average] age in study, body mass index, cigarettes per day, alcohol consumption (drinks) and gender when they were significant covariates (*p *< 0.10). Traditionally, a nonparametric lod score of 3.6 has been used as a genome-wide significance level. However, given our use of seven phenotypes, we adjusted our significance level based on a Bonferroni correction. A LOD score of 3.6 corresponds to a *p*-value of 2.33 × 10^-5^. After correcting for seven tests, the new alpha would be 3.33 × 10^-6^, and so all chromosomal locations with LOD scores greater than 4.4 were identified and prioritized for further evaluation.

## Results

The heritability of the phenotypes were quite high, with estimates greater than 0.60 for the weighted average of all phenotypes except TG. Heritabilities for the phenotypes from a single exam tended to be lower, again except for TG. Results from the genome screen are summarized in Figure 1.

For the longitudinal data (AUC), nine chromosomal regions produced LOD scores greater than 4.4. Linkage (LOD = 28.7) was identified between BMI and chromosome 13, 6 cM from gene G_b11_. The cholesterol phenotype linked to within 2 cM of G_b30 _on chromosome 11 (LOD = 5.5). The highest level of linkage evidence (LOD = 34.6) was found for the fasting glucose phenotype, directly at the G_s3 _gene on chromosome 5. The HDL phenotype linked within 4 cM of G_b12 _on chromosome 9 (LOD = 9.6) and between G_b19 _and G_b20 _on chromosome 17 (LOD = 7.4). Height linked to both G_b1 _on chromosome 5 (LOD = 16.8) and G_b2 _on chromosome 7 (LOD = 6.7). SBP linked to within 3 cM of G_s11 _on chromosome 15 (LOD = 6.4) and to within 7 cM of G_s10 _on chromosome 21 (LOD = 33.8). The longitudinal data for triglycerides did not yield a LOD score above 4.4. But more importantly, there were no false-positive linkage results at this level of significance.

Analyses performed using the cross-sectional study designs yielded consistently lower LOD scores in comparison with the longitudinal measures (see Tables 2 and 3). Using the longitudinal phenotype, linkage to nine different chromosomal regions was detected. Using the less powerful cross-sectional phenotypes, linkage was still detected to many of the same chromosomal regions. When analyzing data from only a single exam (SE), linkage to eight of the nine chromosomal regions was identified, with only the linkage to chromosome 15 for SBP failing to meet our stringent linkage criteria. When employing phenotypic data for individuals collected near age 45 (AGE = 45), linkage to seven of the nine regions was detected and a new linkage for TG was obtained to G_b14 _at the q-terminus of chromosome 1. The two chromosomal regions that were no longer detected were the region on chromosome 15 for SBP and the linkage to chromosome 11 for cholesterol.

Analyses performed using the longitudinal phenotypes, but with the sample size of the cross-sectional data, yielded, as expected, lower LOD scores. However, all genes identified (LOD > 4.4) in the full longitudinal sample were still detected in the smaller longitudinal samples.

## Discussion

Several conclusions can be made from these results. First, it is not surprising that the highest LOD scores were obtained near genes accounting for the greatest percentage of the trait variance. The baseline genes identified by the six highest LOD scores (4.4 ≤ LOD ≤ 28.7) were among the genes accounting for the greatest trait variance (between 15% and 40%). The correlation between these LOD scores and the proportion of phenotypic variance each explained was 0.88 (*p *= 0.02). Even with maximal information from the longitudinal study design and ideal conditions such as complete genotype and phenotype data, two genes contributing a large percentage of the variance to HDL and SBP (20% and 25%, respectively) were not identified. Also, despite this large data set with complete information, we were unable to detect genes with smaller effects. No baseline gene accounting for less than 15% of the trait variance was detected in our analyses. However, the models we utilized also did not identify any false-positive linkage findings.

Second, over 30 values were removed from the GLUC and TG distributions because they were in excess of three standard deviations (with a few above 7 and 10 standard deviations). Removal of the extreme phenotypic outliers reduced the number of false-positive results dramatically (data not shown). By removing these values, the kurtosis of the distribution was reduced from 37.5 to 2.2 and from 21.5 to 1.7 for GLUC and TG, respectively. But while this improved the sensitivity and specificity immensely, GLUC and TG still have the highest levels of kurtosis and are the only measurements with false-positive linkage results. From these data, it appears that it is more important to remove extreme values for the sake of specificity than it is to retain them for power.

Third, when looking at the same phenotype measured cross-sectionally or longitudinally, the heritability of the trait tended to correlate with the magnitude of the linkage signal. This is of particular importance when examining TG levels. The heritability, and hence LOD scores, increased and reached our level of statistical significance only when the subset of subjects with an age near 45 were included in the analysis. By maximizing the heritability of the trait prior to genetic analysis, the power to detect genetic effects was increased.

## Conclusions

Our analyses demonstrated that the use of simple longitudinal phenotypes, AUC, was a powerful means to detect genes of major to moderate effect on trait variability. In a few instances, where the heritability of the trait was increased at a specific age, examining cross-sectional data at a uniform age (TG) provided higher LOD scores and linkage to genes that were not detected using other phenotypic modelling. We conclude that it is important to carefully examine phenotypic traits so as to maximize the ability to detect genes for complex traits. A variety of strategies may be appropriate depending on the situation and the underlying biology. In some instances, a multivariate phenotype that accurately models the underlying biology will provide the most power. However, in most instances, the underlying biology is unknown and assumptions must be made so as to develop phenotypes for genetic analyses. Other strategies to define the phenotypic trait accurately and improve power include identifying the most heritable phenotype, ensuring normality of the trait distribution, and maximizing the information utilized through novel longitudinal designs for genetic analysis.

## Figures and Tables

**Table 1:** Descriptive statistics for the longitudinal phenotypes

**Phenotype**	**Heritability (h^2^)**	**Covariates^A^**	**Proportion of Variance due to Covariates**	**Mean**	**Standard Deviation**	**Variance**
BMI	75.3%	a, g	5.51%	26.54	4.53	20.50
CHOL	64.4%	a, g	4.59%	200.22	30.04	902.38
GLUC	62.6%	a	<0.01%	95.38	13.15	172.92
HDL	65.5%	a, b, c, d, g	26.27%	50.33	11.22	125.88
HEIGHT	76.7%	g	49.46%	65.26	3.94	45.55
SBP	77.7%	a, b, c, g	9.25%	131.32	14.75	217.44
TG	47.4%	a, b, d, g	45.29%	121.45	59.11	3493.90

^A^Significant covariates employed in the linkage analysis (a = average age in study, b = body mass index, c = cigarettes per day, d = drinks, alcohol consumption, g = gender).

**Table 2:** Genome Screen Results for Single Exam (SE)

Phenotype		h^2^	n	covar^A^	1	3	5	7	9	11	13	15	17	19	21	false+
BMI	SE	75.3	2585	a,g	0.6	0.9	1.4	0.9	0.7	1.0	**22.6**	1.0	1.6	0.1	0.0	1.7
	AUC^B^	76.0	2585	a,g	0.6	0.5	1.6	1.2	0.9	1.0	**23.9**	0.9	1.4	0.2	0.0	1.4
Chol	SE	58.6	2550	a,g	0.9	0.8	0.7	1.3	0.8	**5.7**	0.7	2.2	0.4	0.8	0.9	1.4
	AUC	67.7	2550	a,d,g	1.7	0.5	0.1	1.0	2.1	**5.6**	1.1	3.1	0.4	0.4	1.9	1.5
Gluc	SE	50.8	2558	a	3.1	1.5	**20.7**	0.5	0.6	1.3	1.3	0.4	0.1	0.7	0.2	4.7
	AUC	67.1	2558	a	1.8	1.1	**48.8**	0.9	0.2	3.0	1.1	0.0	0.3	3.2	0.0	3.9
HDL	SE	63.8	2565	a,b,c,d,g	1.8	0.5	0.5	1.2	**8.9**	0.5	0.8	0.1	**5.5**	1.0	0.5	2.5
	AUC	65.3	2565	a,c,d,g	1.9	0.9	0.7	0.9	**9.3**	0.7	0.6	0.2	**6.9**	1.3	0.8	2.7
Height	SE	73.7	2585	g	0.2	1.1	**12.3**	**4.7**	0.3	0.2	1.9	0.6	0.3	0.2	0.0	0.7
	AUC	75.0	2585	g	0.4	0.8	**13.8**	**5.1**	0.5	0.2	1.3	1.0	0.6	0.1	0.0	0.9
SBP	SE	57.1	2585	a,b,c	0.1	1.2	2.7	2.8	0.8	0.1	2.9	3.2	1.2	1.3	**19.3**	2.5
	AUC	75.1	2585	a,b,c	0.1	1.7	1.9	2.1	0.5	0.0	1.3	**6.3**	0.4	1.0	**30.6**	2.2
Tg	SE	41.4	2526	a,b,d,g	4.2	0.9	1.5	0.8	2.8	0.5	0.8	0.8	3.7	1.4	0.4	4.5
	AUC	47.2	2526	a,b,d,g	3.9	0.2	3.6	1.0	0.9	0.9	1.2	0.6	1.1	2.0	0.1	1.5

^A^Significant covariates employed in the linkage analysis (a, average age in study; b, body mass index; c, cigarettes per day; d, drinks, alcohol consumption; g, gender). ^B^Results of the longitudinal data with only those individuals included in the cross-sectional design. Only odd chromosomes contain genes; even chromosomes are summarized as highest false positive (false+).

**Table 3:** Genome Screen Results for AGE = 45

Phenotype		h^2^	n	covar.^A^	1	3	5	7	9	11	13	15	17	19	21	false+
BMI	AGE = 45	76.0	2674	g	0.3	0.1	1.1	0.6	1.2	1.3	**20.2**	0.5	1.0	0.2	0.1	1.5
	AUC^B^	76.2	2674	g	0.5	0.2	1.6	1.0	1.1	1.0	**23.0**	1.3	1.2	0.3	0.0	1.7
Chol	AGE = 45	59.7	2650	g	1.6	0.4	0.1	1.0	1.2	3.4	0.9	3.2	0.4	0.3	1.7	1.2
	AUC	66.6	2650	a,g	1.3	0.7	0.1	0.5	0.7	**4.9**	0.6	3.4	0.2	0.3	1.4	1.2
Gluc	AGE = 45	64.6	2412	-	1.0	0.9	**22.2**	0.3	1.9	0.5	0.0	0.1	0.4	0.1	0.1	1.3
	AUC	62.9	2412	a	1.3	1.0	**25.1**	0.3	1.7	1.2	0.0	0.0	0.9	0.3	0.0	3.1
HDL	AGE = 45	65.4	1600	b,c,d,g	2.2	0.5	0.1	1.3	**8.5**	0.4	0.9	0.0	**9.8**	0.5	0.6	2.4
	AUC	66.1	1600	b,c,d,g	2.9	0.8	0.5	1.1	**9.0**	0.2	0.5	0.0	**8.6**	0.7	0.3	2.8
Height	AGE = 45	73.7	2585	g	0.2	1.1	**12.3**	**4.7**	0.3	0.2	1.9	0.6	0.3	0.2	0.0	0.7
	AUC	75.0	2585	g	0.4	0.8	**13.8**	**5.1**	0.5	0.2	1.3	1.0	0.6	0.1	0.0	0.9
SBP	AGE = 45	74.7	2411	a,b,c	0.5	0.8	2.0	1.5	1.1	0.1	1.2	3.6	0.3	1.2	**21.1**	2.1
	AUC	71.3	2411	a,b,c	0.7	2.4	2.7	3.0	0.7	0.0	1.4	**5.3**	0.3	0.8	**26.8**	1.9
Tg	AGE = 45	53.2	1585	a,b,d,g	**6.2**	0.2	0.9	0.7	1.7	1.4	0.3	0.3	0.6	0.3	0.5	1.6
	AUC	51.9	1585	a,b,d,g	**7.6**	0.2	0.7	1.3	2.9	2.5	0.3	0.2	0.6	0.5	0.4	1.1

^A^Significant covariates employed in the linkage analysis (a, average age in study; b, body mass index; c, cigarettes per day; d, drinks, alcohol consumption, g, gender). ^B^Results of the longitudinal data with only those individuals included in the cross-sectional design. Only odd chromosomes contain genes; even chromosomes are summarized as highest false positive (false+).
